# A Qualitative Exploration of Food Insecurity and Institutional Support Among International Students at a Regional Australian University

**DOI:** 10.1002/hpja.70080

**Published:** 2025-07-19

**Authors:** Kwun Ming Leung, Karen Charlton, Anne McMahon, Karen Walton, Rajshri Roy, Katherine Kent

**Affiliations:** ^1^ School of Medical, Indigenous and Health Sciences University of Wollongong Wollongong Australia; ^2^ Discipline of Nutrition and Dietetics, Susan Wakil School of Nursing and Midwifery, Faculty of Medicine and Health The University of Sydney Sydney Australia

**Keywords:** food insecurity, international student, qualitative, university student

## Abstract

**Introduction:**

Food insecurity among international students studying in Australian universities is a growing concern, but limited research has explored their experiences in regional contexts. This study explores food insecurity experiences among international students studying at a regional Australian university, focusing on challenges with food access and the effectiveness of available support services.

**Methods:**

Student‐led focus groups were employed to explore food insecurity experiences among a sample of international students. A semi‐structured guide probed students about food access issues and perceptions of available support services, and student recommendations for improvement were explored. Audio recordings were transcribed, and data were thematically analysed using Braun and Clarke's six‐phase framework.

**Results:**

International students (*n* = 17) participated in four focus groups. Thematic analysis identified challenges affecting students' food security, including inadequate campus food options, the high cost of living, and a lack of culturally appropriate food choices in Australia. Students employed various coping strategies, such as using dietary supplements, meal prepping, and seeking discounted and/or frozen foods. International students encountered significant barriers to accessing support services, including inefficient pantry operations, perceived stigma, and poor communications about options. Based on these findings, participants suggested improvements in pantry management and expanded financial support for university students.

**Conclusions:**

This study highlights the significant challenges and coping strategies related to food insecurity among international students at a regional Australian university.

**So What?:**

Our findings emphasise the urgent need for tailored, culturally appropriate interventions and improved communication strategies that support international students' agency in accessing healthy, appropriate food.

## 1 Introduction

Food security is defined by the United Nations Food and Agricultural Organisation (FAO) as when all people, at all times, have physical and economic access to sufficient safe and nutritious food that meets their dietary needs and food preferences for an active and healthy life [1]. Conversely, food insecurity occurs when these needs cannot be met in socially acceptable ways [1].

Food insecurity has emerged as a growing concern globally, impacting various social, economic, and demographic groups [2]. The 2023 State of Food Security and Nutrition in the World report highlights that nearly one‐third of the global population experienced food insecurity in 2022 [3]. University students are particularly vulnerable, with significantly higher rates of food insecurity than the general population [4]. In Australia, food insecurity rates prior to the COVID‐19 pandemic among university students ranged from 25% to 48% [5, 6]. These rates have since increased to more than half of university students in more recent studies [7, 8], which may be due to the ongoing financial impact of COVID‐19 and subsequent inflation impacting the food supply, limited employment opportunities, and uncertain economic stability [9]. The causes of food insecurity among university students both in Australia and internationally are both numerous and complex, with similarities in the experiences of university students across high‐income countries. This can include housing instability, unemployment, low income, inadequate social support, young age, and poor access to food [2, 10]. The implications of food insecurity extend beyond hunger, impacting academic performance, graduation rates, physical and mental health, university retention, and social integration [2, 4, 11, 12, 13, 14]. Food insecure students are more likely to experience compromised diet quality, academic difficulties such as lack of concentration in class or exams, and poorer physical and mental health [2, 4, 11, 12, 13, 15, 16].

Recent research from a regional Australian university has reported the highest documented food insecurity rate in Australia of 54% [15], with a higher proportion of international students (63%) found to be food insecure compared to domestic students (50%) [15]. International students are considered to be at higher risk of food insecurity as a result of unique challenges they face in transitioning to living in Australia, including language barriers, higher tuition costs, and lack of family support, as well as having limited access to culturally appropriate foods [2, 17, 18, 19]. Many are employed in precarious, low‐paid casual jobs with limited job security, and often reside in housing located far from campus, increasing both transport and time burdens. They often lack access to familial or community support, and research suggests that international students and their families may underestimate the challenges of food insecurity when planning to study abroad [20]. The role of universities in providing services and having strategies in place to address food insecurity is therefore essential to support educational outcomes and promoting student well‐being, particularly for international students [21].

To address food insecurity, some universities have implemented various initiatives, including food pantries or food banks (where students can receive emergency food relief in the form of emergency groceries or meals), community gardens, and scholarships to assist students purchasing basic necessities such as food [22]. Food pantries are among the most widely used approaches [22]. However, for university food support initiatives to be effective, several barriers need to be overcome, including stigmatisation experienced by students and logistical challenges [23, 24, 25]. Stigma often arises from a perception of failure, where students feel judged for using food assistance services [25]. Qualitative research indicates that students, including international students, often avoid using these services due to embarrassment, shame, and fear of judgement [26]. Logistical barriers, such as inconvenient opening hours and locations, further make food aid less accessible to those students who need it, especially those with conflicting class schedules [24, 27, 28]. Some research has identified that pantries do not stock sufficient culturally appropriate foods, while other studies have reported that international students are ineligible to receive food relief at some universities [29].

Despite these barriers, other research shows that students can benefit from support services and food insecurity initiatives. Indeed, when they are eligible to access food relief, international students have been shown to be more frequent users of university campus food pantries than domestically enrolled students [30]. A review article suggested that successful food security initiatives require coordinated efforts in marketing and operations thereof to raise awareness without stigmatising food insecure students. Users' dignity should also be considered by providing flexible access and an adequate amount of hygienic and safe food products [31]. Supporting cultural food access for international students should also be a priority. Continuous improvement processes that monitor user satisfaction can further enhance programme effectiveness [31]. Additionally, universities should consider incorporating food literacy education, such as nutrition education, budgeting, and cooking workshops, into programme curricula. Higher food literacy is associated with long‐term benefits even when campus‐based interventions are not available [22].

Despite ongoing research on food security among university students, there remains a notable gap in qualitative research that directly captures international students' voices on the effectiveness of university support services in these settings. Understanding how students perceive and experience these resources is essential to developing targeted strategies to address food insecurity in this demographic [32]. Most studies rely on quantitative data, overlooking the nuanced insights that qualitative approaches can offer [4]. Additionally, food insecurity experiences vary significantly across different university environments, and there has been limited research focused on regional Australian universities, despite a high prevalence of food insecurity. This potentially overlooks the unique challenges and needs of those attending regional institutions where cultural, economic, and logistical factors may differ significantly from metropolitan contexts. There is a need for context‐specific studies to gain insights that can inform responsive and effective support initiatives tailored to this context [33]. Therefore, the objectives of this study are to use a series of student‐led focus groups with international students to (i) explore food access issues and associated impacts on international students, (ii) assess the effectiveness of current university support services addressing food insecurity, (iii) gather student‐informed recommendations for improving existing and future support initiatives.

## 2 Methods

### 2.1 Study Context

With a student population of approximately 35 000, the University of Wollongong is a regional Australian university with its main campus located in Wollongong. This study was conducted at University of Wollongong, which operates nine campuses across Australia. As of 2024, there were approximately 27 000 onshore students, with around 30% being international students. These students represent a diverse range of countries, primarily from Southern and Central Asia, and Southeast Asia. Recent research from University of Wollongong reported the highest documented rate of food insecurity at an Australian university, with 54% of students experiencing food insecurity [15].

In University of Wollongong's new financial inclusion strategy ‘increasing access to healthy food’ has been identified as a priority action area in University of Wollongong, which was developed to respond to growing student financial issues and provide a roadmap for supporting vulnerable university students. In particular, the university has identified the need to increase the amount of food relief provided to students which is an important step towards addressing food insecurity. However, exploring student perspectives on food insecurity and the universities responses is still an important step in shaping future service delivery.

### 2.2 Study Design

This study employed a qualitative design to gather in‐depth data on international students' experiences with food insecurity. Focus groups were conducted to explore students' perspectives on food access challenges, the effectiveness of existing university support services, and recommendations for future improvements.

### 2.3 Sampling, Participant Recruitment, and Ethical Considerations

We used a broad sampling approach to recruit international students aged 18 years and older who self‐identified as experiencing food insecurity to ensure a diverse inclusion of students from different academic disciplines, demographics, and levels of study. Recruitment was carried out by advertising the flyer through University of Wollongong student organisations and university communication channels, such as newsletters, social media platforms, and printed flyers. Interested students were provided with the Participant Information Sheet and a Consent Form. Prior to the focus group, participants completed an online Qualtrics survey to provide demographic information and assess their food insecurity status using the USDA 6‐item food security screening tool [34]. We used the USDA 6‐item Food Security Survey Module to reduce participant burden and ensure consistency with previous food insecurity research conducted at the university. All participants received a $30 gift card as recognition for their contribution. This study was approved by the University of Wollongong Human Research Ethics Committee (HREC number 2024/025). All participants provided informed consent for participation, including consent for their discussions to be audio‐recorded.

### 2.4 Data Collection Tools and Procedures

Based on their preference and availability, participants were grouped into focus sessions with 2–6 individuals, with each group meeting for approximately 1 h. Focus group sessions were held either face‐to‐face or via Zoom, depending on participant preference. The discussions were semi‐structured, guided by a pre‐developed focus group guide that covered key topics such as food insecurity experiences, university support services, and potential solutions to improve food insecurity. Focus groups were selected to encourage rich, co‐generated insights into students' experiences. While the potential for shame around food insecurity may inhibit discussion, this was mitigated by using student‐led groups in familiar campus settings, creating a safe and conversational environment.

Discussions were audio‐recorded and facilitated by an experienced researcher or trained student researcher who was trained to mitigate potential bias. Facilitator training focused on fostering a respectful, inclusive environment and mitigating potential bias by using neutral language, active listening, and strategies to encourage participation from all group members. The focus group guide was informed by current literature on food insecurity, with questions designed to elicit information on participants' lived experiences, coping strategies, and views on improving campus food insecurity. Participants were encouraged to provide recommendations for enhancing existing university support services.

### 2.5 Data Analysis

Descriptive statistics were summarised to represent the demographic characteristics of participants. The variables included age, gender, year of study, and education level. Food security status was calculated using the USDA 6‐item food security screening tool [34], previously adopted in research into food insecurity in Australian university students [35, 36]. The audio recordings from the focus groups were transcribed using Otter AI (Otter.ai, 2023), and transcriptions were manually reviewed for accuracy. Thematic analysis was conducted following Braun and Clarke's six‐phase framework to ensure a rigorous and systematic approach to data interpretation [37]. NVivo software (QSR International, 2020, Version 12) was utilised to manage data and facilitate an inductive coding process. The analysis began with reading and re‐reading the transcripts to document initial ideas and identify preliminary codes. Initial codes were then generated by systematically coding features across the dataset. Similar responses were collated under each code, allowing for the identification of patterns across the dataset. Recurrent themes were then searched by bringing relevant codes together. Authors (KML and KK) then discussed and refined the initial themes, developing a preliminary thematic framework, which was followed by several rounds of refinement with ongoing discussions with the broader team. Consensus was reached after any differing views were mediated through discussion with the team. Once the final themes were generated, each theme was carefully defined and given an informative description. Interactions between the themes were considered. A narrative synthesis supported by quotes was then written to present the results, ensuring that the final themes accurately reflected the data.

## 3 Results

A total of 17 international students completed the study (Table 1). Of all participants, 23.5% (*n* = 4) reported experiencing marginal food insecurity, 41.2% (*n* = 7) moderate food insecurity, and 29.4% (*n* = 5) severe food insecurity, while 5.9% (*n* = 1) were classified as food secure. Participants represented a range of nationalities, including Chinese (29.4%), Vietnamese and Hong Kong students (17.6% each), and Japanese and Mongolian students (5.9% each), with 11.7% of participants not reporting their nationality. The sample was predominantly female (64.7%), with most participants aged between 25 and 34 years (53.0%). The remaining participants were either aged 18–24 years (35.3%) or 35 years and older (11.7%). In terms of the year of study, over half of the participants were in their first year (58.8%), with smaller proportions in their second (23.5%), third (11.7%), or fourth year (5.9%). Regarding education levels, 47.0% of participants were pursuing undergraduate degrees, and 53.0% were pursuing postgraduate degrees.

**TABLE 1:** Demographic statistics.

Demographic category	Number of participants	Percentage (%)
Sample size	17	100.0
Age distribution (years)		
18–24	6	35.3
25–34	9	53.0
35+	2	11.7
Gender		
Male	6	35.3
Female	11	64.7
Year of study		
First year	10	58.8
Second year	4	23.5
Third year	2	11.7
Fourth year	1	5.9
Nationalities		
Sri Lankan	2	11.7
Chinese	5	29.4
Vietnamese	3	17.6
Hong Kong	3	17.6
Japanese	1	5.9
Mongolian	1	5.9
Not reported	2	11.7
Student education level		
Undergraduate	8	47.0
Postgraduate	9	53.0
Food insecurity status		
Food secure	1	5.9
Marginal insecure	4	23.5
Moderate insecure	7	41.2
Severely insecure	5	29.4

**FIGURE 1:**
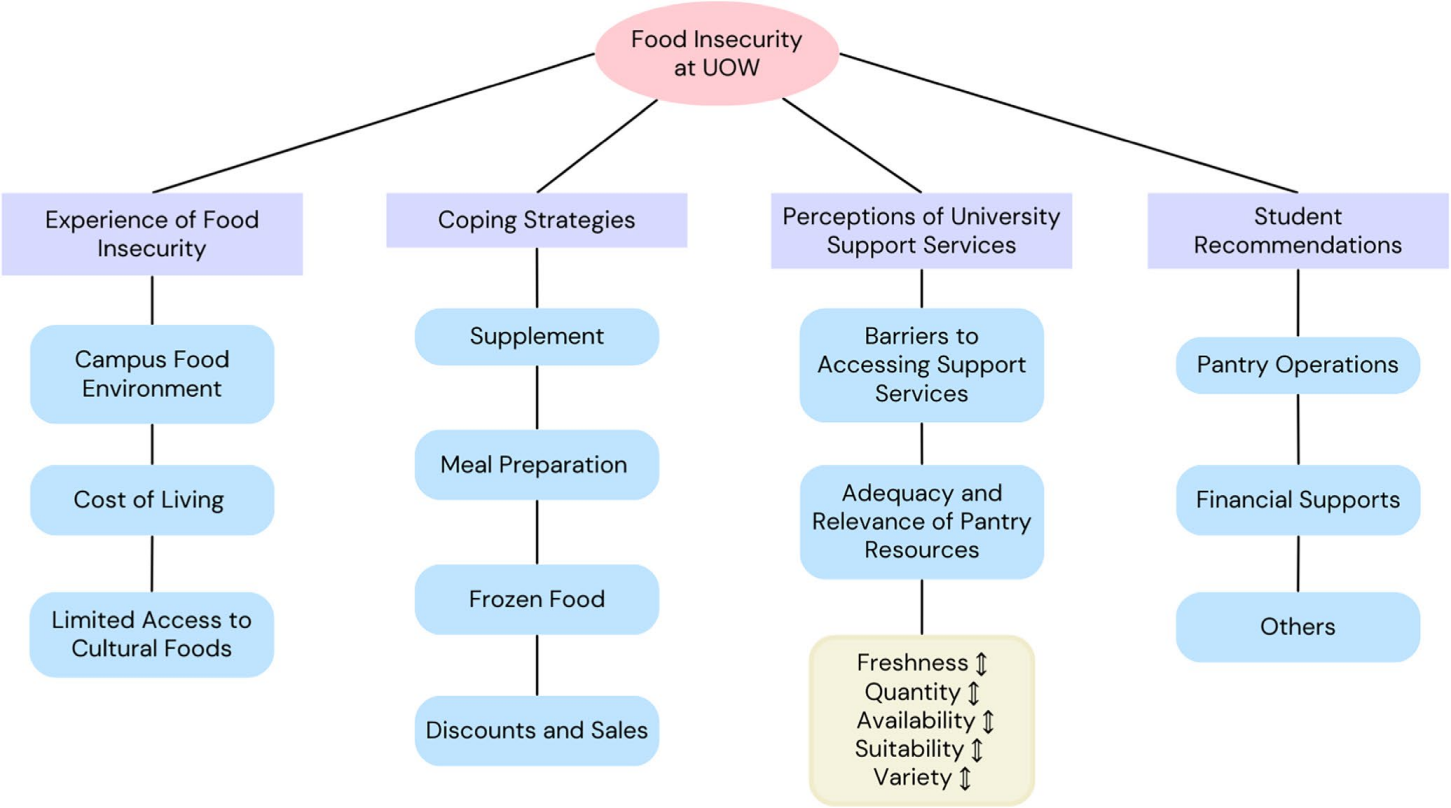
Overview of key themes.

The thematic analysis revealed four key themes, with a summary overview provided in Figure 1. The themes included (i) Experience of food insecurity; (ii) Coping strategies; (iii) Perceptions of university support services, and (iv) Student recommendations. These themes have various subthemes that are described in more detail in a narrative synthesis below.

## 4 Theme I: Experience With Food Insecurity

### 4.1 Campus Food Environment

Students perceived campus food prices to be higher than off‐campus alternatives. They usually avoid on‐campus purchases unless necessary. One participant noted:

They [the university supermarkets] charge a price that has a premium on top … making it very expensive compared to the market … I seldom buy it from them. As for the restaurant or those places that sell food in canteen… again is overpriced. So I tend not to buy unless I'm very hungry or needed. (#1)

This sentiment resonated with other students who described restaurants and cafes on campus as overpriced. Most students often prefer eating off campus where ‘the price is a little bit cheaper and the taste is much better’. (#2) Additionally, students feel resigned that campus food providers do not offer a *‘student price’*: ‘we don't really have a student price, it's actually more expensive than outside’ (#2).

The limited operating hours of campus supermarkets and restaurants were another significant concern, particularly for students with busy schedules or those wishing to dine on campus later in the day. Several food outlets close as early as 2:30 pm, restricting students' access to meals later in the day. One student noted: ‘The closing time is so early … Chinese restaurant on campus close at maybe 2:30 … you have no choice to eat dinner on campus’ (#3). These early closing times limit meal options for students outside typical business hours.

### 4.2 Cost of Living

Many students reported that the high cost of living, particularly food expenses, restricted their ability to prepare nutritionally balanced or culturally familiar meals. Financial constraints often made it challenging for students to prioritise healthy eating, with one student noting that: ‘If you want a balance meal or a healthy meal … you can't do that on a budget’ (#1). This was especially true for cultural foods or ingredients, with one student saying ‘I used to add a lot of spices. But .. in Australia … the cost of spices are really high …, it cost me a lot’ (#7). Social interactions were also affected by these financial constraints, with one participant noting their inability to join classmates for meal: ‘I need to make my own meal, [I] can't go to Ugly Duckling [a restaurant on campus] with them, sitting under the sun, that's cute but I cannot’ (#8). Additionally, high rent costs further strained students' budgets, pushing some to reduce food expenses to accommodate other financial responsibilities.

### 4.3 Limited Access to Cultural Foods

The cultural food options on campus were discussed, with students stated that the available dishes do not align with their taste preferences: ‘The tofu here is so sweet that I cannot stand, especially the Mapo tofu. It is too sweet so I don't like it’ (#3). This sentiment was resonated by other students. Some students reported that the availability of culturally specific foods or certain foods was another concern. Certain ingredients that are readily available in their home country were either unavailable or difficult to find in Australia. ‘We have fried noodles, and the noodles are usually pre‐packed back at home … but here, there's no same thing to replace it, so I have to make it by hand’ (#10). Another student said: ‘I'm from Bangladesh, that [type of food is] my favourite, you know, very unavailable. And if they want to buy, costly’ (#11). This scarcity often requires students to make adjustments or to invest additional time in preparing meals.

## 5 Theme II: Coping Strategies

Students adopted various coping strategies to manage food insecurity they experienced. One common approach was the use of dietary supplements to compensate for nutritional deficiencies, especially when access to healthy foods is limited. For some, this provides both practical and psychological relief. ‘Sometimes I think I eat food not very healthy … and I will take some supplement … maybe to support my health’ (#12). Similarly, other students reported using supplements as a form of reassurance: ‘I also sometimes buy supplements like vitamins because … it's more like a placebo effect to make me feel like I'm eating healthy’ (#9).

Meal preparation was another strategy frequently mentioned strategy to manage the high cost of living: ‘If I have time, the night before I would pack my lunch, for example, some spaghetti or some simple fruit’ (#2). By doing meal preparation, students may avoid the higher costs of eating out. Financial pressures also influenced the types of food students could afford. Frozen foods emerged as a convenient and cost‐effective alternative to fresh produce, as one student explained: ‘When I first came to Australia, I wasn't accustomed to the price difference between groceries here and back home, so I sometimes resort to frozen pre‐made stuff’ (#9).

In addition to meal preparation, students actively sought discounts and sales to reduce food expenses. Some wait until late afternoon to access discounted meals, while others bought items close to their best‐before dates to save money: ‘One of my colleagues and sometimes I would buy discounted foods, one day past or near the best‐before date’ (#13).

## 6 Theme III: Perceptions of University Support Services

### 6.1 Barriers to Accessing Support Services

This theme encompasses six key areas that students may face difficulties in accessing support services, including time costs, queue system, frequency of access, location, staffing, and awareness and promotion of available services. These barriers collectively contribute to students' perceptions of university support services.

### 6.2 Time Cost

One main barrier to accessing university support services is the time cost. Students often struggled to balance accessing these resources with their academic commitments. One participant explained: ‘I try to [access the pantry] but like, I have class at 8:30 on Wednesday, [and] even it says it will start at eight, it never starts at eight’ (#10). Another student shared the difficulty of managing extended waiting times and the stigma of accessing support: ‘One time because of the long wait time I was late for class, and then I ended up carrying like a bag of groceries to my tutorial, and it was a bit weird’ (#9). These experiences demonstrate the difficulty students face not only in managing their time but also in navigating the potential stigma of accessing support services.

### 6.3 Queue System

The queue system for the food pantry was another commonly noted barrier. Students expressed frustration with the current ‘lucky draw’ method to determine the service order. One student explained: ‘The line‐up system is a lucky draw system on Thursday, which is not reasonable at all… it makes it really hard to estimate the time needed’ (#5). Another participant commented: ‘The ticket number and the called number are both random. So, this is too random’ (#3). The randomness of the system made it challenging for students to plan their schedules effectively around their academic and personal schedules.

### 6.4 Frequency of Access

There were mixed opinions regarding the frequency of access. While students felt that the current frequency was ‘definitely not enough’ (#1), expressing a desire for more regular opportunities to access food, others noted that the weekly food pantries weekly limit of food items allowed per student (10 food items) meant that increasing access would not necessarily increase the amount of food they could redeem. One student explained: ‘It can be open every day, but everyone is only allowed to get the goods here one time’ (#3).

### 6.5 Location

Students expressed mixed opinions regarding the location of the pantry. Some students felt that the pantry located near the university entrance made it accessible to all: ‘I mean, being located, like right in front of the Uni library makes it easy for everyone to know where it is’ (#9). However, others felt uncomfortable or self‐conscious about the pantry located in such a prominent space. One student even described the experience as deeply stigmatising: ‘I feel so bad when I stand there… I am begging my food in front of everyone… it's just emotionally not very comfortable’ (#8).

### 6.6 Staff

Feedback regarding the staff were generally positive, with students acknowledging their patience and understanding: ‘Staff is good, very patient’ (#14). Some students recognised the challenges staff faced due to limited resources: ‘You can tell with the limited resources; they probably have limited funds to provide for people that need it’. This recognition underscores the complexity of delivering adequate support within a constrained resource environment.

### 6.7 Awareness and Promotion of Support Services

A significant barrier to accessing university support services was the perceived lack of promotion and awareness. While many participants were familiar with some initiatives, awareness of other support services was low. One student remarked: ‘Even though they have all the support, most students don't know about them. There's no promotion advertising about it’. Another student reflected on how she only recently discovered the food pantry: ‘I just came to know about Pantry this January’ (#11). This suggests that communication efforts may not be reaching all students, particularly those who are not frequently on campus. Some participants also highlighted non‐university programmes, such as ‘Fun Food Friday’ organised by a religious group, which may be more accessible to certain students.

### 6.8 Adequacy and Relevance of Pantry Resources

This theme encompasses five key aspects: freshness, quantity, availability, suitability, and variety of the food provided by the university pantry, which are all interconnected, often influencing one another (Figure 1).

### 6.9 Freshness (Quality and Shelf Life)

Some students expressed concerns regarding the freshness of food provided by the food pantry, as some items are close to their expiration dates. One participant mentioned: ‘Food [in Pulse pantry] is not so fresh… some of the food… may be very close to the expiry date’ (#3). Another student expressed frustration: ‘You find some things, grab that and go to the home and after that you check to find, ‘Oh, it's out of date’ (#14). Despite these concerns, some students were willing to overlook expiration dates, prioritising access over freshness, with one student shared: ‘I don't care about the expiry day, I will eat it’ (#8). This illustrates the compromises students are willing to make in the face of food insecurity.

### 6.10 Quantity (Point System)

The weekly 10‐point system allowing students to redeem food items was generally perceived as fair given the pantry's limited resources. One student commented: ‘I feel like for the points system … is quite reasonable’ (#9). While the system aims to promote equitable access, some participants expressed that the number of points could occasionally fall short of their needs. One student stated: ‘Sometimes we feel that we don't have enough points, and sometimes okay, [depending on the week]’ (#15). This indicates that while the system generally worked, its effectiveness was associated with the availability of desirable items.

### 6.11 Availability

The lack of essential items, such as rice, eggs, milk, and vegetables, emerged as a frequent concern. Many students reported that these items are often depleted. One participant shared: ‘When it is your turn, basically everything is gone’ (#3). Another stated: ‘when I go to this [the food pantry], vegetables was gone… I can't get any vegetable from the pantry’ (#16). Long wait times exacerbated the issue, with students waiting ‘an hour and a half’ only to find that ‘a lot of the items will be gone and you'll be left with the things that you don't want’ (#1). This scarcity affected students' perceptions of both availability and the suitability of remaining options.

### 6.12 Suitability of Food

The pantry was generally valued as a resource to ease financial strain. However, some items, such as sauces and seasonings, were seen as less useful. When staple foods like bread and vegetables ran out, students were left with less suitable options. This aspect is closely related to the availability of suitable food options.

### 6.13 Variety

The variety of food was another area where opinions diverged. Some felt the selection was limited, with one participant stating: ‘The variety is very limited… maybe 10 things to choose, give or take’ (#1). Conversely, other students appreciated the options: ‘I think the variety is quite good’ (#9). These differing opinions suggest a nuanced relationship between variety and availability, with students' experiences influenced by the timing of their visits and the availability of popular items.

### 6.14 Theme IV: Student Recommendations

Students offered various recommendations to improve the campus food pantry and other support services. Opinions on the pantry's queue system were divided, some advocated for a ‘first come, first serve’ approach (#11), while others raised concerns over security. Some students suggested alternative systems such as online ticketing or reservations. In terms of location, students expressed a desire for multiple pantry sites to improve privacy, with one student noted: ‘It would be better to set up some other places’ (#14). Frequency of operation was also highlighted, with several students suggesting more flexible hours to accommodate different schedules. Students also recommended more effective advertising strategies, including specifying pantry offerings in advance and using varied communication platforms to reach a broader audience. In terms of food offerings, there were calls for greater variety, quality control, and an increased availability of essential items to reduce disappointment for those at the end of the queue. Additionally, students suggested that the university provide more financial support, such as concessions on campus food and vouchers for essentials. Some students also proposed initiatives such as gardening programmes, which could offer both sustainability and experiential learning. Collectively, these recommendations point to a need for improved operational efficiency, increased accessibility, and enhanced support to promote food security on campus.

## 7 Discussion

This study offers crucial insights into the specific challenges and coping mechanisms related to food insecurity among international students at University of Wollongong, underlining the need for tailored university support services. Key challenges include limited availability of culturally relevant food and high costs within the campus environment. Students adopted coping strategies including using dietary supplements, preparing meals, and seeking discounts to manage their budgets. Students' perceptions of support services highlighted barriers such as operational inefficiencies and a lack of awareness, identifying clear areas for policy and intervention improvements.

Participants consistently cited high prices, limited operating hours, and a lack of culturally relevant options as major obstacles in the campus food environment. This supports findings from other studies indicating widespread dissatisfaction among food insecure students with campus food affordability [5]. Existing university policies fail to effectively regulate food prices or ensure affordability, inadequately supporting students' needs. An audit of food environments across nine Australian universities ranked the University of Wollongong 8 out of 9 in terms of governance aligned with the provision of equitable and healthy food options on campus [38]. Australian universities should consider partnerships with campus food vendors to provide subsidised, affordable meal options specifically for students identified as food insecure, thereby addressing both affordability and accessibility in a resource‐limited context.

Participants also reported restricted operating hours and a lack of culturally relevant options as further barriers to food security. Food outlets closed early in the day, preventing students from obtaining meals during evening hours or following late classes. This is consistent with a study that suggested that flexible operating hours are an important factor influencing students' decisions in choosing a dining outlet [39]. Although vending machines are available for after‐hours access, they primarily offer snacks that are not aligned with the Australian Dietary Guidelines [40]. Extending operating hours or incorporating nutritious options in vending machines might mitigate this barrier and improve accessibility. International students often seek culturally familiar foods as a key component of maintaining cultural identity and for enjoyment of recognisable dishes from their home countries [41]. In our sample of international students, the taste differences in food from their home country were a strong consideration that prevented them from purchasing more food on campus. Universities could address this gap by engaging international students in the menu development process or conducting regular feedback sessions for a culturally inclusive menu.

The international students in our study adopted various coping strategies to manage food insecurity, such as buying discounted or near‐expiry foods, preparing meals in advance, and supplementing their diet with vitamins, which are similar to other research [42, 43, 44]. These behaviours highlight the economic pressures students face and highlight their resourcefulness in navigating these challenges. These findings align with previous studies that show similar adaptive behaviours among food insecure students [28, 42, 43, 45, 46]. This illustrates the precarious nature of students' food security, as they prioritise affordability over nutritional value and potentially food safety, often leading to poorer dietary quality and potential long‐term health implications [42]. A previous University of Wollongong study also indicated that food insecure students often compromise on diet quality [15]. The use of dietary supplements as a coping strategy is also noteworthy, as it highlights that some students may recognise nutritional gaps in their diet but are unable to afford healthier options. While supplements may offer some relief, they are not a sustainable solution to food insecurity, as they do not address the underlying issue of inadequate access to nutritious food. This finding underscores the need for comprehensive support programmes that focus not only on food provision but also on nutrition education and food literacy to facilitate making healthy, balanced options.

Despite appreciating the university's support services, students reported significant access barriers such as conflicting academic schedules, inefficient queuing systems, and poor visibility of these services. These barriers show that there is a need for the university to streamline access and enhance the visibility of support mechanisms. Time constraints, particularly those associated with students' academic schedules, operational delays, and lengthy wait times were a significant barrier to utilising services such as the campus food pantry. This finding is consistent with prior research that has shown time issues can discourage students from accessing food assistance programmes [47]. A more flexible or streamlined approach to accessing food support services, such as online reservations or pre‐packed food parcels, as suggested by participants, may mitigate these barriers. Some students also reported perceived stigma associated with using food support services. This reflects a profound sense of vulnerability associated with publicly accessing food assistance, which may deter students from seeking help even when needed. Previous research indicates concerns about being seen using such services can significantly deter students from accessing available resources [25, 47]. Relocating the food pantry to a more discreet yet accessible location or locations could reduce these concerns and encourage more students to utilise the services. A lack of awareness and promotion of support services further complicates the situation. Despite university efforts to offer food assistance, many students were unaware of the full range of resources available. This indicates a significant communication gap, as the presence of university support services did not translate to effective utilisation. The findings highlight the need for improved visibility and outreach, as many students remained unaware of services that could support their food needs. This lack of awareness has been similarly reported in other studies, where insufficient outreach efforts impact students' access to food assistance programmes [25, 27]. There is a clear need for more effective communication strategies. A targeted campaign utilising multiple communication channels, such as the student association, social media platforms, and email, might improve student awareness of available resources and encourage greater use.

## 8 Strengths, Limitations and Future Research

The qualitative design of this study allowed for an in‐depth exploration of participants' experiences, capturing insights often overlooked in quantitative research. The focus group approach also captured students' voices, empowering them to actively contribute to service improvement on campus. While this study fills a critical gap in the literature by focusing specifically on international students, several limitations should be acknowledged. First, as all participants were international students, the findings are not generalisable to the broader student population, particularly domestic students, who may experience food insecurity differently due to greater access to familial, social, or government support systems. Although prior research has more commonly examined domestic student experiences, the lack of domestic student participation in this study prevents a comparative analysis and limits the applicability of findings beyond this specific cohort. Additionally, the qualitative design may have introduced social desirability bias, particularly during face‐to‐face discussions, potentially resulting in the underreporting of certain behaviours or reliance on food support services. Although facilitators were trained to mitigate bias, interviewer influence could still have impacted the depth and direction of responses, as participants might have tailored their responses to align with perceived expectations. Furthermore, the study involved a relatively small sample size. While data saturation was achieved, which has been confirmed through an information power approach where no new themes or concepts emerged in the final focus group, the limited sample size restricts the generalisability of the findings to other university settings. Future studies could address these limitations by employing a mixed‐methods approach, incorporating quantitative surveys to capture a broader range of experiences to provide a more comprehensive understanding of food insecurity in higher education and support the development of inclusive, evidence‐based interventions.

## 9 Conclusion

This study provides valuable insights into the complex issue of food insecurity among international students at a regional Australian university. While the study focused on international students, many of the identified coping strategies and barriers are likely shared by other food‐insecure students. This suggests that interventions designed to address student food insecurity may have broad relevance and benefit the wider student population. Our findings emphasise an urgent need for interventions that address food affordability, accessibility and the cultural appropriateness of food options, as well as considering operational aspects that improve the effectiveness of support services. Specifically, based on this study, universities should prioritise not only the expansion of culturally appropriate food options but also improve the affordability and accessibility of these foods. Enhancing support services through education on nutrition and cost‐effective cooking, and forging partnerships with local food suppliers who can offer a diverse range of affordable food choices, are essential steps. By involving international students in the development and evaluation of these services, the university can ensure that the solutions implemented are well‐suited to meet their specific needs, fostering a supportive and inclusive campus environment. By continuing to adapt and respond to these needs, the university can set a standard for inclusivity and support for international students.

## Consent

Informed consent was obtained from all subjects involved in the study. This study was approved by the University of Wollongong Human Research Ethics Committee (HREC number 2024/025).

## Conflicts of Interest

The authors declare no conflicts of interest.

## Data Availability

The data that support the findings of this study are available from the corresponding author upon reasonable request.
